# Cold-induced urticaria associated with type I cryoglobulinemia, successfully treated with rituximab

**DOI:** 10.1016/j.jdcr.2021.10.032

**Published:** 2021-11-11

**Authors:** Leonie C. Stroet, Maud A.W. Hermans, M. Maurer, Martijn B.A. van Doorn

**Affiliations:** a Department of Dermatology, Urticaria Center of Reference and Excellence (UCARE), Erasmus Medical Center, Rotterdam, The Netherlands; b Department of Internal Medicine, Section of Allergy & Immunology, Urticaria Center of Reference and Excellence (UCARE), Erasmus Medical Center, Rotterdam, The Netherlands; c Dermatological Allergology; Department of Dermatology and Allergy, Charité, Urticaria Center of Reference and Excellence (UCARE), Universitätsmedizin, Berlin, Germany; d Department of Allergology and Immunology, Fraunhofer Institute for Translational Medicine and Pharmacology ITMP, Berlin, Germany

**Keywords:** cold-induced urticaria, cryoglobulinemia, inducible urticaria, rituximab, ColdU, cold-induced urticaria, Ig, immunoglobulin

## Introduction

Cold-induced urticaria (ColdU) is a common form of chronic inducible urticaria. It is characterized by the development of wheals and, sometimes, angioedema upon skin exposure to cold air, liquids, or objects.^1^ Although considerable progress has been made over the last years in the understanding of the condition and its treatment, ColdU remains a challenging clinical problem. Patients often suffer from ColdU for years before they are diagnosed, and treatment requires a personalized approach. Similar to other forms of chronic urticaria, ColdU has a significant impact on quality of life.^1^ Here, we describe a case of severe and refractory ColdU associated with cryoglobulinemia and successfully treated with rituximab.

## Case report

**Fig 1:**
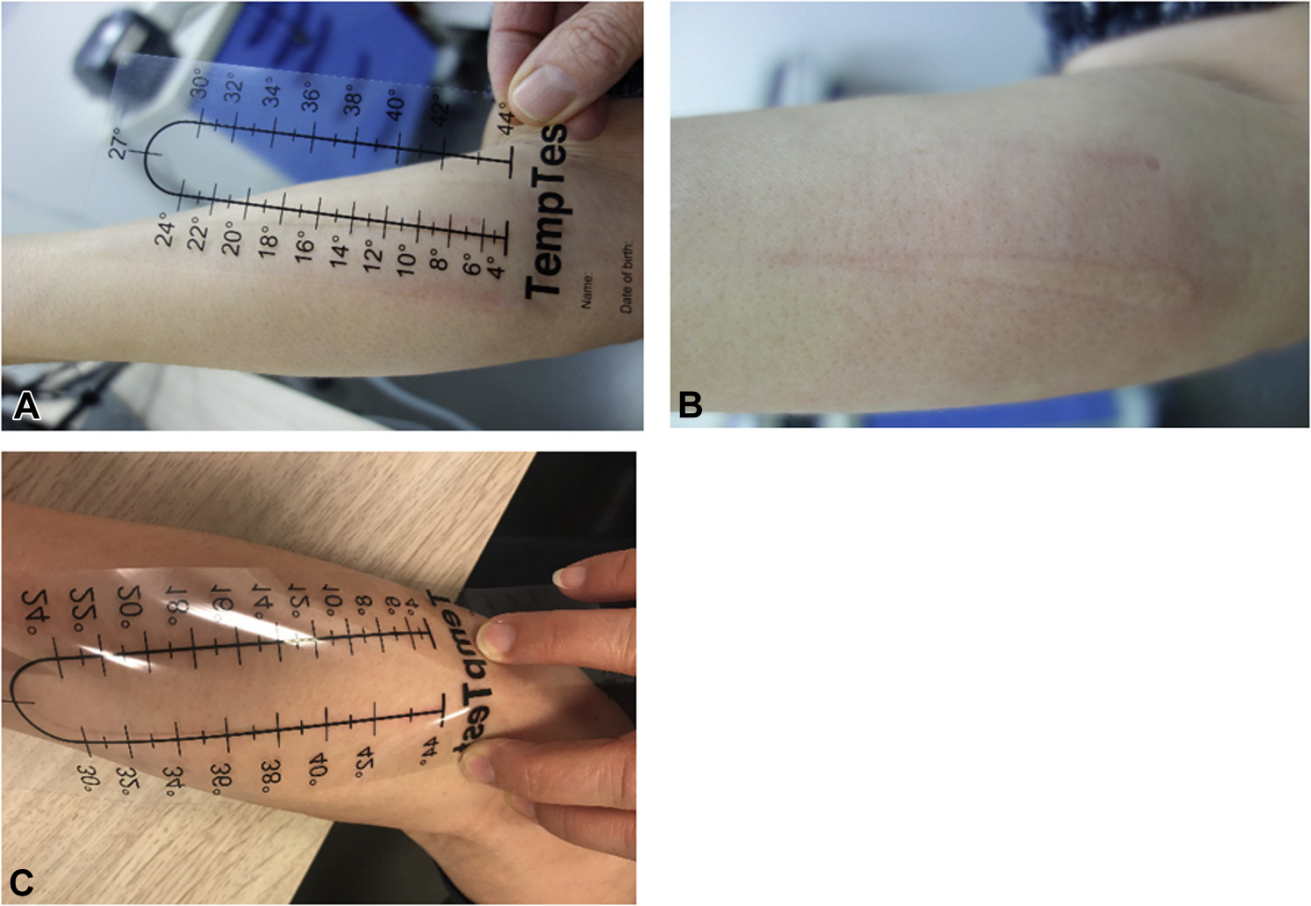
TempTest results at baseline (T_0_) showed a clear wheal-and-flare reaction between 4 °C (**A**) and 16-18 °C (**B**). Three months after rituximab therapy, the TempTest was negative (**C**).

A 60-year-old woman was referred to our Urticaria Center of Reference and Excellence^2^ because of ColdU, from which she suffered since the age of 30. Localized pruritic wheals occurred when the ambient temperature was below approximately 18 °C for longer than 3-4 minutes, upon exposure to cold wind, or when touching cold objects. A cold provocation test (TempTest) was positive at a temperature of 18 °C and lower (Fig 1). The dermatology life quality index was 16, indicating a large impact on her quality of life.

Her general medical history included hypertension and monoclonal B-cell lymphocytosis. The latter was diagnosed 7 years prior to presentation. A wait-and-see policy was maintained, since lymphocyte counts were stable during follow-up. The family history was negative for chronic urticaria and auto-inflammatory diseases. Treatment with at least 3 different H1-antihistamines at up to 4-fold of the standard dose, omalizumab dosed up to 600 mg every 4 weeks subcutaneously, and cyclosporin up to 5 mg/kg was unsuccessful. Because of the severe and refractory nature of her ColdU, further investigations were performed. This revealed type I cryoglobulins (monoclonal IgG), which were associated with the monoclonal B-cell lymphocytosis. No mutations associated with auto-inflammatory conditions were found with whole exome sequencing.

We hypothesized that, in this patient, the cryoglobulins detected were of pathophysiologic importance for her ColdU and started treatment with rituximab, a therapeutic monoclonal antibody directed against CD20 that reduces autoantibody production via depletion of memory B-lymphocytes. The patient received 2 intravenous administrations of 1000 mg rituximab with a 2-week interval. Three months later, the patient reported a remarkable improvement of her symptoms. She could now walk outside at a temperature of 8 °C for 3 hours and was able to touch cold objects without developing signs or symptoms of ColdU. Also, the cold provocation test was now negative (Fig 1), the lymphocyte count normalized, and the cryoglobulin level decreased to 0.05 g/L (Table I). At the time of writing, the complete response was still maintained 7 months after rituximab treatment, and cryoglobulin levels were decreased further, to 0.02 g/L.

**Table I:** Laboratory results before and after treatment with rituximab

Lab test [reference values/intervals]	Before rituximab	3 months after rituximab	7 months after rituximab
Cryoglobulins [<0.03 g/L]	0.18 g/L, type I monoclonal IgG	0.05 g/L	0.02 g/L
IgE [<100 kU/L]	8	-	-
IgA [0.76-3.9 g/L]	0.67	0.71	0.73
IgG [7.0-16.0 g/L]	10.7	8.9	7.0
IgM [0.45-2.30 g/L]	0.27	0.25	0.27
C-reactive protein [<10 mg/L]	3.9	-	4.3
Erythrocyte sedimentation rate [0-30 mm/h]	17	-	-
Hemoglobin [7.5-9.5 mmol/L]	7.9	7.9	8.2
Leukocytes [3.5-10.0 × 10^9^/L]	12.0	6.0	6.6
Lymfocytes [15%-50%]	73.9	37.2	36.9

*Ig*, Immunoglobulin.

## Discussion

In this report we describe a case of complete clinical remission of ColdU associated with type I cryoglobulinemia after treatment with rituximab in a patient with monoclonal B-cell lymphocytosis.

Cryoglobulins are immunoglobulins that precipitate at temperatures below 37 °C. Precipitation can cause occlusion of vessels, sometimes leading to immune-complex vasculitis and tissue damage. Cryoglobulins can be classified into 3 types according to clonality and type of immunoglobulin. Type 1 consists of monoclonal immunoglobulins, mostly IgG or immunoglobulin M and is mostly seen in clonal B-cell diseases. Type 2 describes a mixture of monoclonal immunoglobulin M and polyclonal IgG and is associated with autoimmune diseases and hepatitis C infection. A mixture of polyclonal immunoglobulin M and IgG can be classified as type 3 cryoglobulins. Dermatological findings in patients with cryoglobulinemia can include skin purpura, necrotic ulcers, cold-induced acrocyanosis, and the Raynaud phenomenon.^3^

ColdU is a very rare manifestation of cryoglobulinemia, and the pathophysiology has not yet been elucidated. Our patient had classical, cursory wheals matching urticaria rather than vasculitis, which would be expected in typical cryoglobulinemic skin lesions. Potentially, the cryoglobulins in this particular case are matching an epitope on mast cells and basophils, causing IgG mediated degranulation.^4^ Unfortunately, we did not perform a basophil or mast cell activation test, mostly because of the technical difficulties of working with cryoglobulins *in vitro*. However, the striking effect of B-cell depletion with rituximab corroborates the hypothetical presence of autoreactive immunoglobulins in this patient, and the clear association with ColdU suggests a pathophysiologic role for the cryoglobulins.

Although rare, cryoglobulinemia should be considered in refractory ColdU. In a large French cohort study, 5 of 104 patients had cryoglobulinemia,^5^ and a recent systematic review and meta-analysis of 14 relevant studies with a total of 1151 ColdU patients showed that 3.0% (19/628) of patients had detectable levels of cryoglobulins.^6^ Furthermore, we identified 2 case reports of relevance to our case. One described a 13-year-old boy with hepatitis B-associated type 2 cryoglobulinemia who developed ColdU. Upon clearance of the hepatitis B infection, his ColdU disappeared together with the cryoglobulins.^7^ The other published case concerned a patient with cryoglobulinemia associated with chronic lymphocytic leukemia and ColdU as the only clinical manifestation. The patient was treated with chlorambucil 10 mg once daily for 6 weeks, followed by 2 mg/day maintenance and steroids. Hematologic remission ensued, and the ColdU improved, although complete remission was not achieved.^8^ Lastly, in a cohort of 35 patients with ColdU, 46% had detectable cold agglutinins, and 27% had cryoglobulins.^9^ None of these studies explored the potential of rituximab as treatment for cryoglobulin-associated ColdU.

Rituximab is a monoclonal antibody that targets B-cells and has a position in the treatment of both autoimmune disorders; eg, rheumatoid arthritis and hematologic diseases.^10^ The use of rituximab for ColdU is off-labeled and was administered in the setting of an academic hospital after multiple on-label treatments had proven to be unsuccessful. Off-label use of rituximab should at any time be carefully considered, as rituximab may provide serious adverse events. Infusion reactions are most common and can range from headache and fever up to bronchospasm, hypotension, and, in rare cases, anaphylaxis-like reactions.^11^

In conclusion, cryoglobulinemia may underlie some cases of ColdU. The role of cryoglobulins in the pathophysiology of ColdU has not yet been elucidated in detail and requires further investigation. Until then, we propose testing for cryoglobulinemia in patients with refractory ColdU, and B-cell depleting therapy can be considered when cryoglobulins are detected.

## Conflicts of interest

None disclosed.
